# Use of silver nanoparticles increased inhibition of cell-associated HIV-1 infection by neutralizing antibodies developed against HIV-1 envelope proteins

**DOI:** 10.1186/1477-3155-9-38

**Published:** 2011-09-18

**Authors:** Humberto H Lara, Liliana Ixtepan-Turrent, Elsa N Garza Treviño, Dinesh K Singh

**Affiliations:** 1 Department of Life Sciences, Winston-Salem State University, Winston Salem, NC, 27110, USA; 2 Laboratorio de Terapia Celular, Departamento de Bioquimicay Medicina Molecular, Facultad de Medicina Universidad Autonoma de Nuevo Leon, Mexico

**Keywords:** Silver Nanoparticles, Neutralizing Antibodies, HIV, gp120, gp41

## Abstract

**Background:**

HIV/AIDS pandemic is a worldwide public health issue. There is a need for new approaches to develop new antiviral compounds or other therapeutic strategies to limit viral transmission. The envelope glycoproteins gp120 and gp41 of HIV are the main targets for both silver nanoparticles (AgNPs) and neutralizing antibodies. There is an urgency to optimize the efficiency of the neutralizing antibodies (NABs). In this study, we demonstrated that there is an additive effect between the four NABs and AgNPs when combined against cell-associated HIV-1 infection *in vitro*

**Results:**

Four NABs (Monoclonal antibody to HIV-1 gp41 126-7, HIV-1 gp120 Antiserum PB1 Sub 2, HIV-1 gp120 Antiserum PB1, HIV-1 gp120 Monoclonal Antibody F425 B4e8) with or without AgNPs of 30-50 nm in size were tested against cell free and cell-associated HIV_IIIB _virus. All NABs inhibited HIV-1 cell free infection at a dose response manner, but with AgNPs an antiviral additive effect was not achieved Although there was no inhibition of infection with cell-associated virus by the NABs itself, AgNPs alone were able to inhibit cell associated virus infection and more importantly, when mixed together with NABs they inhibited the HIV-1 cell associated infection in an additive manner.

**Discussion:**

The most attractive strategies to deal with the HIV problem are the development of a prophylactic vaccine and the development of effective topical vaginal microbicide. For two decades a potent vaccine that inhibits transmission of infection of HIV has been searched. There are vaccines that elicit NABs but none of them has the efficacy to stop transmission of HIV-1 infection. We propose that with the addition of AgNPs, NABs will have an additive effect and become more potent to inhibit cell-associated HIV-1 transmission/infection.

**Conclusions:**

The addition of AgNPs to NABs has significantly increased the neutralizing potency of NABs in prevention of cell-associated HIV-1 transmission/infection. Further exploration is required to standardize potentiation of NABs by AgNPs. It is also required to evaluate in vivo toxicity of AgNPs before AgNPs could be incorporated in any antiviral vaginal creams.

## Introduction

The pandemic of Acquired Immunodeficiency Syndrome (AIDS), caused by the Human Immunodeficiency Virus Type 1 (HIV-1) infection, is a worldwide public health issue [1]. The latest estimates by the Joint United Nations Program on HIV/AIDS (UNAIDS) indicate that more than 33.3 million people worldwide are living with HIV-1 infection or AIDS.

The medical use of the cocktail drugs known as highly active antiretroviral therapies (HAART) has significantly reduced morbidity and mortality among AIDS patients [2,3]. Unfortunately, the achievement of HAART is insufficient and compromised by the evolution of drug resistance HIV strains [4]. Consequently, the search for new therapies to inhibit viral infection or to restore the damaged immune system in HIV/AIDS patients continues. Newly discovered drugs are constantly evaluated as therapeutic drug candidates. These new drugs are eagerly awaited for the growing number of HIV-infected individuals who have developed resistance to the currently existing antiretrovirals [5].

The most attractive strategies to deal with the HIV problem are the development of a prophylactic vaccine and the development of an effective topical vaginal and rectal microbicides. Both approaches are essential and eventually a combination of the two may prove to be most effective strategy in controlling the HIV-1 epidemic by diminishing the incidence of human-to-human transmission events [6].

The discovery of an HIV-1 vaccine that elicits broadly efficient neutralizing antibodies still remains an elusive goal especially after the recent failure of the leading T cell based HIV vaccine in human efficacy trials [7]. The envelope glycoproteins gp120 and gp41 that are the main targets for neutralizing antibodies are partially shielded by N-linkedglycans and other structurally-imposed steric constraints that limit antibody access to potential neutralization epitopes. The complex level of antigenic diversity of HIV-1, the shielding of key epitopes within the three dimensional structure of the native Env trimer, and the failure of newer versions of Env proteins to elicit broadly reactive antibodies have led to some pessimism regarding the potential to ever elicit high titers of neutralizing antibodies against diverse strains of HIV-1. Therefore there is a need to maximize the efficiency of whatever titers of neutralizing antibodies generated by vaccines [8].

A significant correlation is usually reported linking the ability of an antibody to neutralize HIV-1 *in vitro *and to protect *in vivo *against HIV-1 in animal models. Some vaccine research studies have measured the capability of specific NABs to protect against SHIV infection, and found that efficient immunity is achieved only when the serum concentration of NABs in the challenged animals is many multiples of the *in vitro *neutralization titer. Normally these NABs require relatively high antibody concentrations that may be highly difficult to reach by vaccination [9].

Silver ions in complexes or compounds have been used for centuries to disinfect fluids, solids and tissues [10]. There is no cross resistance with antibiotics [11] and probably there is also no induction of antimicrobial resistance by silver ions [12]. The Crede's solution (silver nitrate 0.2%) has been used to prevent the Neonatal conjunctivitis *("ophtalmia neonatorum") *which is a form of bacterial conjunctivitis contracted during delivery. The eyes are infected during passage through the birth canal from a mother infected with either Neisseria gonorrhoeae or Chlamydia trachomatis. Crede's solution was used to prevent the condition. If left untreated it could cause blindness [13]. Also Silver sulfadiazine is widely used by physicians to treat severe burns in skin, this topical cream not only acts against infections, but also against inflammation and enhance the healing of the tissue. The many attempts to find a better remedy for the topical treatment of burns than silver sulphadiazine have so far been without success [14].

Recent advances in nanotechnology have enabled the scientific community to investigate and manipulate materials at nanometer level. Nano-based delivery systems can be adapted to modulate drug release, reduce drug-associated toxicity, protect drugs from metabolism, and target drugs to affected cells, tissues, and compartments [15-19]. Nowadays we can use pure silver of nanometer sizes. We previously reported that AgNPs inhibit HIV-1 and that these nanoparticles attach to the gp120 [20]. Then we investigated the mode of antiviral action, with a panel of tests we probed that AgNPs:- a) attach to the envelope of the HIV-1 inhibiting the interaction with CD4 receptor,:-b) inhibits a wide range of HIV-1 regardless of the tropism,:-c) inhibit entry and fusion of the virus to the target cell at a non-toxic range. AgNPs proved to be more efficient than silver ions at non-cytotoxic levels [21].

With the above antiviral characteristics, AgNPs are appealing to be included as an active compound in a vaginal topical gel. We previously demonstrated that Polyvinylpyrrolidone (PVP) AgNPs mixed in a topical gel, inhibit the transmission of infection when applied to the human cervical tissue in a model for explants, at a non-toxic range, and more significantly, AgNPs acts rapidly in less than a minute and protect the human cervical tissue for more than 48 hours even after an extensive wash of the gel, without any toxicity to the human cervical explants [22].

In the present study we decided to investigate the additive effect of AgNPs with four NABs (Monoclonal antibody to HIV-1 gp41 126-7 [23], HIV-1 gp120 Antiserum PB1 Sub 2, HIV-1 gp120 Antiserum PB1 [24-26], HIV-1 gp120 Monoclonal Antibody F425 B4e8 [27]) as both act against viral envelope glycoprotein trimers on the surface of the virus that mediate receptor binding and entry.

## Results

### Inhibition of cell free HIV_IIIB _virus infection by Monoclonal antibody to HIV-1 gp41 (126-7) and Silver Nanoparticles in U373-MAGI-CXCR4_CEM _cells

**Figure 1:**
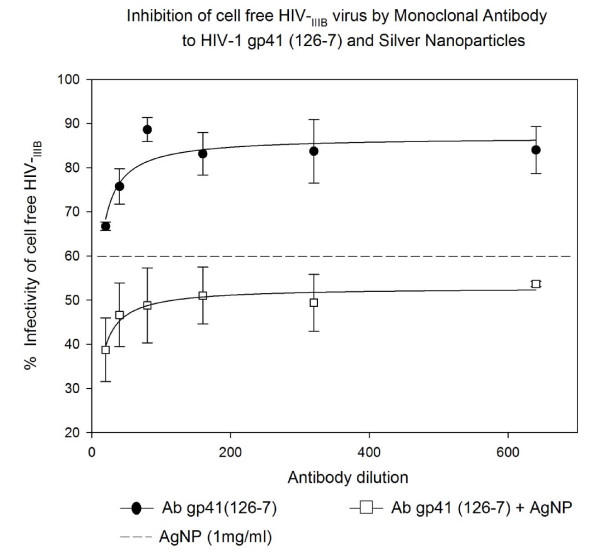
**HIV inhibition of cell free HIV_IIIB _virus infection by Monoclonal antibody to HIV-1 gp41 (126-7) and Silver Nanoparticles**. Serial two-fold dilutions of Monoclonal antibody to HIV-1 gp41 (126-7) were added to 10^5 ^TCID_50 _of HIV-1_IIIB _cell-free virus. After incubation for 5 minutes, they were added with or without silver nanoparticles at 1 mg/mL. Then the mixture was placed into 96-well plates with indicator cells (U373-MAGI-CXCR4_CEM_) at a final 0.2-0.5 m.o.i. Assessment of HIV-1 infection was made with a luciferase-based assay. The percentage of residual infectivity after treatment was calculated with respect to the positive control of untreated virus. The assay was performed in triplicate; the data points represent the mean, and the solid lines are nonlinear regression curves done with SigmaPlot 10.0 software. By means of Mann- Whitney Rank Sum test we compared the difference in the median values between the two groups (only AgNPs and AgNPs with antibody to HIV-1 gp41 126-7) is greater than would be expected by chance; there is a statistically significant difference (P < 0.002).

In this experiment, we evaluated inhibition of cell free HIV-1_IIIB _virus infection by monoclonal antibody to HIV-1 gp41 (126-7) in U373-MAGI-CXCR4_CEM _cells. The toxic dose of 1 mg/ml AgNPs was ascertained on a cytotoxicity assay and was found to be 28% (data not shown). The AgNPs alone showed 40% inhibition of cell free HIV-1_IIIB _virus infection at this concentration against a control (virus infection wihout AgNPs). The monoclonal antibody to HIV-1 gp41 (126-7) alone showed ability to inhibit infection (16-25%) of HIV-1_IIIB _in a dose response manner. The different dilutions of NAB, when added with AgNPs at 1 mg/mL, increased HIV-1_IIIB _inhibition by 47-63% (P < 0.002) until the NAB dilution of 1:160. There was no additive effect observed.(Figure 1).

### Inhibition of cell free HIV_IIIB _virus infection by HIV-1 gp120 Antiserum (PB1) and Silver Nanoparticles in U373-MAGI-CXCR4_CEM _cells

**Figure 2:**
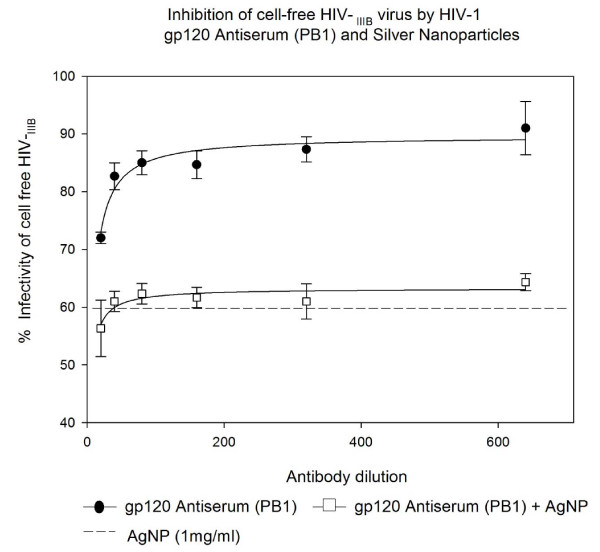
**HIV inhibition of cell free HIV_IIIB _virus infection by HIV-1 gp120 Antiserum (PB1) and Silver Nanoparticles**. Serial two-fold dilutions of HIV-1 gp120 Antiserum (PB1) was added to 10^5 ^TCID_50 _of HIV-1_IIIB _cell-free virus. After incubation for 5 minutes, they were added with or without silver nanoparticles at 1 mg/mL. Then the mixture was placed into 96-well plates with indicator cells (U373-MAGI-CXCR4_CEM_) at a final 0.2-0.5 m.o.i. Assessment of HIV-1 infection was made with a luciferase-based assay. The percentage of residual infectivity after treatment was calculated with respect to the positive control of untreated virus. The assay was performed in triplicate; the data points represent the mean, and the solid lines are nonlinear regression curves done with SigmaPlot 10.0 software. By means of Mann- Whitney Rank Sum test we compared the difference in the median values between the two groups (only AgNPs and AgNPs with antibody to HIV-1 gp120 Antiserum PB1) is not great enough to exclude the possibility that the difference is due to random sampling variability; there is not a statistically significant difference (P < 0.065).

The HIV-1 gp120 Antiserum (PB1) alone showed inhibition of HIV-1 _IIIB _infection in a dose response manner (10-30%). The addition of AgNPs at 1 mg/mL showed no effect in this experiment. The HIV-1 gp120 Antiserum (PB1) dilutions 1:20 and 1:40 showed mild inhibition alone when compared to the inhibition by the mixture of AgNPs and NABs (47 and 41% inhibition, P < 0.065). After that dilution inhibition of HIV-1 _IIIB _virus decreased and was less than AgNPs alone 40%, (Figure 2).

### Inhibition of cell free HIV_IIIB _virus infection by HIV-1 gp120 Antiserum (PB1 Sub 2) and Silver Nanoparticles in U373-MAGI-CXCR4_CEM _cells

**Figure 3:**
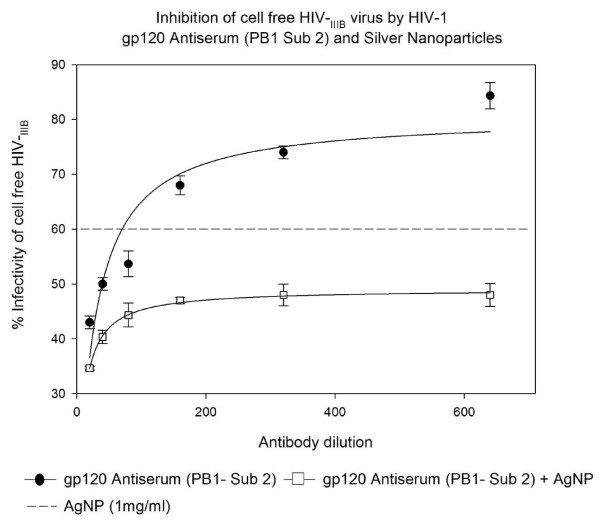
**HIV inhibition of cell free HIV_IIIB _virus infection by HIV-1 gp120 Antiserum (PB1 Sub 2) and Silver Nanoparticles**. Serial two-fold dilutions of HIV-1 gp120 Antiserum (PB1 Sub 2) was added to 10^5 ^TCID_50 _of HIV-1_IIIB _cell-free virus. After incubation for 5 minutes, were added with or without silver nanoparticles at 1 mg/mL. Then the mixture was placed into 96-well plates with indicator cells (U373-MAGI-CXCR4_CEM_) at a final 0.2-0.5 m.o.i. Assessment of HIV-1 infection was made with a luciferase-based assay. The percentage of residual infectivity after treatment was calculated with respect to the positive control of untreated virus. The assay was performed in triplicate; the data points represent the mean, and the solid lines are nonlinear regression curves done with SigmaPlot 10.0 software. By means of Mann- Whitney Rank Sum test we compared the difference in the median values between the two groups (only AgNPs and AgNPs with antibody to HIV-1 gp120 Antiserum PB1 Sub 2) is greater than would be expected by chance; there is a statistically significant difference (P < 0.008).

The HIV-1 gp120 Antiserum (PB1 sub 2) alone was found to have the best ability to inhibit infection of HIV-1_IIIB _(18-71%) in a dose response manner compared to other three NABs. When added with AgNPs at 1 mg/mL, an increase of inhibitory effect was observed until the NAB dilution of 1:640. The addition of AgNPs increased HIV-1_IIIB _inhibition (42-72%, P < 0.008). there was no additive effect. (Figure 3).

### Inhibition of cell free HIV_IIIB _virus infection by HIV-1 gp120 Monoclonal Antibody (F425 B4e8) and Silver Nanoparticles in U373-MAGI-CXCR4_CEM _cells

**Figure 4:**
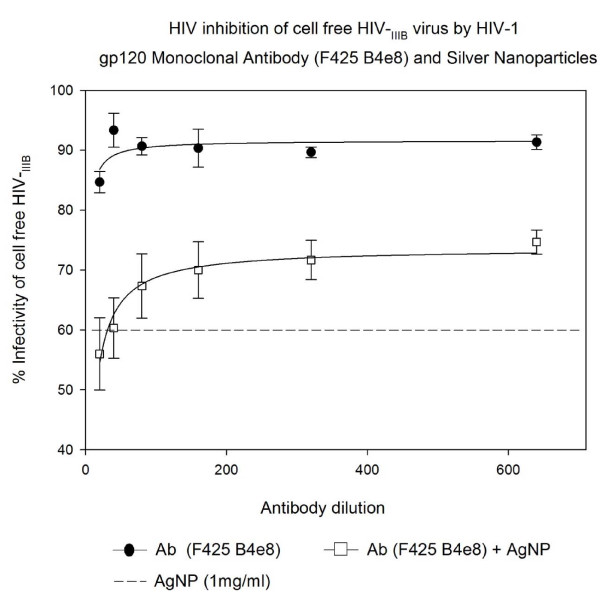
**HIV inhibition of cell free HIV_IIIB _virus infection by HIV-1 gp120 Monoclonal Antibody (F425 B4e8) and Silver Nanoparticles**. Serial two-fold dilutions of HIV-1 gp120 Monoclonal Antibody (F425 B4e8) was added to 10^5 ^TCID_50 _of HIV-1_IIIB _cell-free virus. After incubation for 5 minutes, they were added with or without silver nanoparticles at 1 mg/mL. Then the mixture was placed into 96-well plates with indicator cells (U373-MAGI-CXCR4_CEM_) at a final0.2-0.5 m.o.i. Assessment of HIV-1 infection was made with a luciferase-based assay. The percentage of residual infectivity after treatment was calculated with respect to the positive control of untreated virus. The assay was performed in triplicate; the data points represent the mean, and the solid lines are nonlinear regression curves done with SigmaPlot 10.0 software. By means of Mann- Whitney Rank Sum test we compared the difference in the median values between the two groups (only AgNPs and AgNPs with antibody to HIV-1 gp120 Monoclonal Antibody F425 B4e8) is greater than would be expected by chance; there is a statistically significant difference (P < 0.008).

The HIV-1 gp120 Monoclonal Antibody (F425 B4e8) was found to mildly inhibit infection of HIV-1_IIIB _in a dose response manner (5-11%). When added with AgNPs at 1 mg/mL no effect was observed. The use of AgNPs along with HIV-1 gp120 Monoclonal Antibody (F425 B4e8) showed inhibition efficacy of NAB 36-40% (P < 0.008) which was less than AgNPs alone (Figure 4).

### Inhibition of cell associated HIV_IIIB/H9 _virus infection by Monoclonal antibody to HIV-1 gp41 (126-7), and Silver Nanoparticles in U373-MAGI-CXCR4_CEM _cells

**Figure 5:**
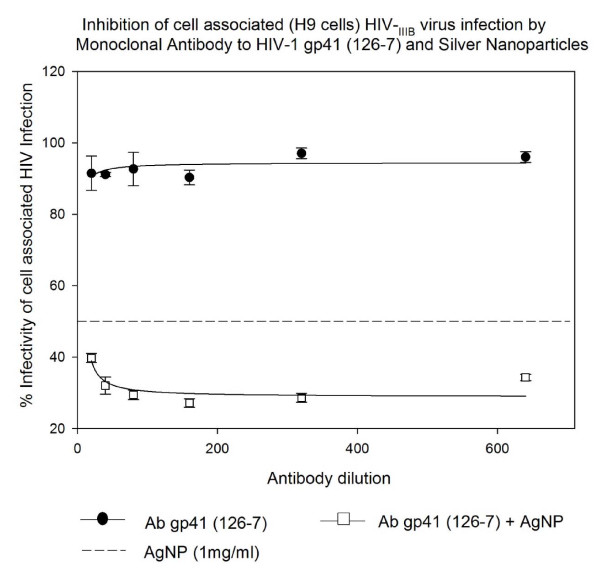
**HIV inhibition of cell associated HIV_IIIB _virus infection by Monoclonal antibody to HIV-1 gp41 (126-7) and Silver Nanoparticles**. Chronically HIV-1-infected H9 (10^5 ^cells) were incubated with serial two-fold dilutions of HIV-1 gp120 Antiserum (PB1) for 5 minutes with or without silver nanoparticles at 1 mg/mL. Then treated H9 cells were placed into 96-well plates with indicator cells (U373-MAGI-CXCR4_CEM_). Assessment of HIV-1 infection was made with a luciferase-based assay after 48 hours. The assay was performed in triplicate; the data points represent the mean, and the solid lines are nonlinear regression curves done with SigmaPlot 10.0 software. By means of Mann- Whitney Rank Sum test we compared the difference in the median values between the two groups (only AgNPs and AgNPs with antibody to HIV-1 gp41 126-7) is greater than would be expected by chance; there is a statistically significant difference (P < 0.002).

The monoclonal antibody to HIV-1 gp41 (126-7) itself has very little effect (6-10% inhibition) on cell associated HIV-1_IIIB/H9 _virus infection. The AgNPs however showed inhibition of HIV-1_IIIB/H9 _virus infection (50%) at 1 mg/mL concentration. The monoclonal antibody to HIV-1 gp41 (126-7) when added with AgNPs showed additive effect till 1:640 dilutions, increasing inhibition of HIV-1_IIIB/H9 _virus to 62-71% (P < 0.002). This inhibitory effect was however lost after 1:640 dilution of NAB and only the inhibition of AgNPs alone were observed (Figure 5).

### Inhibition of cell associated HIV_IIIB/H9 _virus infection by HIV-1 gp120 Antiserum (PB1) and Silver Nanoparticles in U373-MAGI-CXCR4_CEM _cells

**Figure 6:**
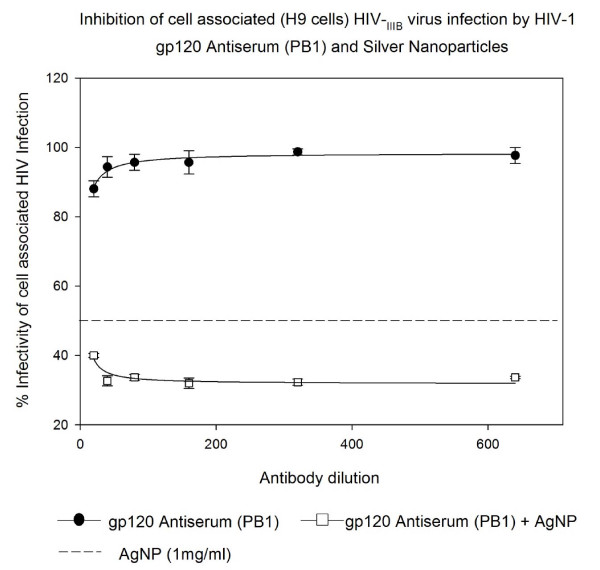
**HIV inhibition of cell associated HIV_IIIB _virus infection by HIV-1 gp120 Antiserum (PB1) and Silver Nanoparticles**. Chronically HIV-1-infected H9 (10^5 ^cells) were incubated with serial two-fold dilutions of Monoclonal antibody to HIV-1 gp41 (126-7) for 5 minutes with or without silver nanoparticles at 1 mg/mL. Then treated H9 cells were placed into 96-well plates with indicator cells (U373-MAGI-CXCR4_CEM_). Assessment of HIV-1 infection was made with a luciferase-based assay after 48 hours. The assay was performed in triplicate; the data points represent the mean, and the solid lines are nonlinear regression curves done with SigmaPlot 10.0 software. By means of Mann- Whitney Rank Sum test we compared the difference in the median values between the two groups (only AgNPs and AgNPs with antibody to HIV-1 gp120 Antiserum PB1) is greater than would be expected by chance; there is a statistically significant difference (P < 0.002).

The HIV-1 gp120 Antiserum (PB1) showed 3-12% inhibitory effect on cell associated HIV-1_IIIB/H9 _virus, addition of AgNPs at 1 mg/mL increased inhibition of virus (60-68% inhibition, P < 0.002) suggesting a strong additive effect of AgNPs on HIV-1 gp120 Antiserum-PB1(Figure 6).

### Inhibition of cell associated HIV_IIIB/H9 _virus infection by HIV-1 gp120 Antiserum (PB1 Sub 2) and Silver Nanoparticles in U373-MAGI-CXCR4_CEM _cells

**Figure 7:**
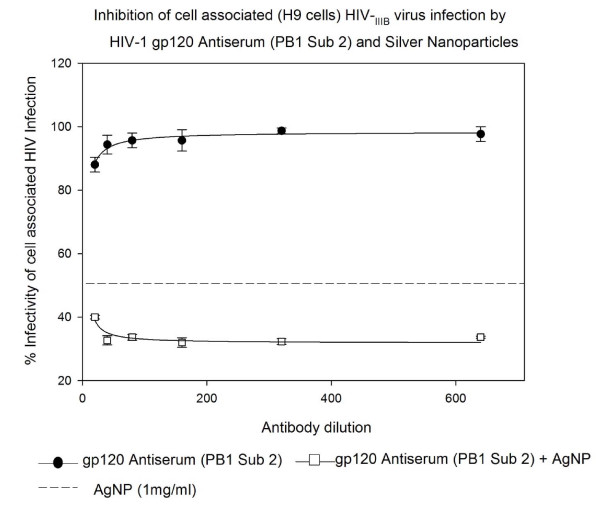
**HIV inhibition of cell associated HIV_IIIB _virus infection by HIV-1 gp120 Antiserum (PB1 Sub 2) and Silver Nanoparticles**. Chronically HIV-1-infected H9 (10^5 ^cells) were incubated with serial two-fold dilutions of Monoclonal antibody to HIV-1 gp120 Antiserum (PB1 Sub 2) for 5 minutes with or without silver nanoparticles at 1 mg/mL. Then treated H9 cells were placed into 96-well plates with indicator cells (U373-MAGI-CXCR4_CEM_). Assessment of HIV-1 infection was made with a luciferase-based assay after 48 hours. The assay was performed in triplicate; the data points represent the mean, and the solid lines are nonlinear regression curves done with SigmaPlot 10.0 software. By means of of Mann- Whitney Rank Sum test we compared the difference in the median values between the two groups (only AgNPs and AgNPs with antibody to HIV-1 gp120 Antiserum PB1 Sub 2) is greater than would be expected by chance; there is a statistically significant difference (P < 0.002).

The HIV-1 gp120 Antiserum (PB1 sub 2) alone showed 3-12% inhibition of cell associated HIV-1_IIIB/H9 _virus infection in a dose response manner. This inhibition was increased to 61-69% inhibition (P < 0.002) when added with AgNPs at 1 mg/mL concentration indicating an additive effect between AgNPs and HIV-1 gp120 Antiserum-PB1 Sub 2 (Figure 7).

### Inhibition of cell associated HIV_IIIB/H9 _virus infection by HIV-1 gp120 Monoclonal Antibody (F425 B4e8) and Silver Nanoparticles in U373-MAGI-CXCR4_CEM _cells

**Figure 8:**
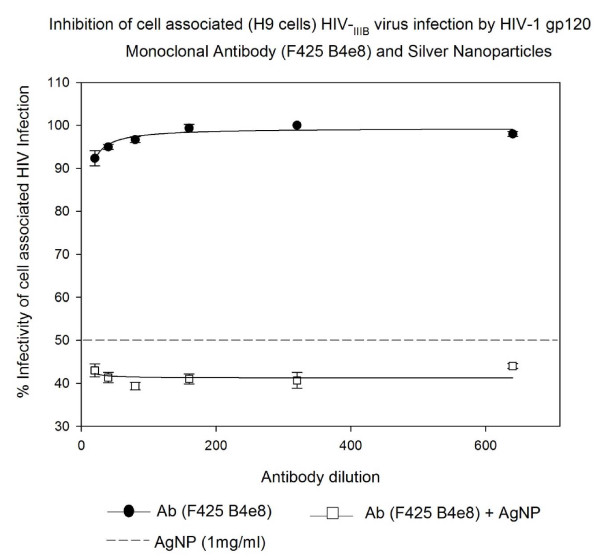
**HIV inhibition of cell associated HIV_IIIB _virus infection by HIV-1 gp120 Monoclonal Antibody (F425 B4e8) and Silver Nanoparticles**. Chronically HIV-1-infected H9 (10^5 ^cells) were incubated with serial two-fold dilutions of HIV-1 gp120 Monoclonal Antibody (F425 B4e8) for 5 minutes with or without silver nanoparticles at 1 mg/mL. Then treated H9 cells were placed into 96-well plates with indicator cells (U373-MAGI-CXCR4_CEM_). Assessment of HIV-1 infection was made with a luciferase-based assay after 48 hours. The assay was performed in triplicate; the data points represent the mean, and the solid lines are nonlinear regression curves done with SigmaPlot 10.0 software. By means of Mann- Whitney Rank Sum test we compared the difference in the median values between the two groups (only AgNPs and AgNPs with antibody to HIV-1 gp120 Monoclonal Antibody F425 B4e8) is greater than would be expected by chance; there is a statistically significant difference (P < 0.002).

The HIV-1 gp120 Monoclonal Antibody (F425 B4e8) alone showed 1-9% inhibition of cell associated HIV-1_IIIB/H9 _virus infection in a dose response manner. Addition of AgNPs at 1 mg/mL concentration resulted in significant increase (P < 0.002) in the inhibitory effect of this cocktail signifying an additive effect between AgNPs and HIV-1 gp120 Monoclonal Antibody (F425 B4e8). The inhibition of cell associated HIV-1_IIIB/H9 _virus infection increased to 58-60% (Figure 8).

## Discussion

Vaccine-induced neutralizing antibodies that inhibit viral entry or fusion to the target cell are the protective correlates of most existing HIV vaccines [[8,9] and [23]]. Nevertheless, for highly variable viruses such as HIV-1, the ability to elicit broadly neutralizing antibody responses through vaccination has proven to be extremely difficult.

The major targets for HIV-1 NABs are the viral envelope glycoprotein trimers on the surface of the virus that mediate receptor binding and entry [24,27]. HIV-1 has evolved many mechanisms on the surface of envelope glyco-proteins to evade antibody-mediated neutralization, including the masking of conserved regions by glycan, quaternary protein interactions and the presence of immunodominant variable elements. In our previous studies we have demonstrated that silver nanoparticles also bind to gp120 and gp41 part of HIV-1 envelop to inhibit HIV-1 infectivity [21,22].

The silver nanoparticles and NABs both use epitopes on the HIV-1 envelope glycoproteins as their binding targets. It was important to study if they could increase HIV-1 inhibition when used together. In our previous studies, we had reported toxicity and dose dependent inhibition of HIV-1_IIIB _by silver nanoparticles [21,22]. In the present study, we have used most effective but least toxic concentration of silver nanoparticles [21] to evaluate its effect on neutralizing ability of four NABs against cell free HIV-1_IIIB _and cell associated HIV-1_IIIB/H9 _virus in U373-MAGI-CXCR4_CEM _cells. In the first experiment, we evaluated inhibition of cell free HIV-1_IIIB _virus infection by monoclonal antibody to HIV-1 gp41 (126-7), HIV-1 gp120 antiserum (PB1), HIV-1 gp120 antiserum (PB1 sub 2), HIV-1 gp120 monoclonal antibody (F425B4e8), and compared that with HIV-1 inhibition by AgNPs alone and relevant NABs + AgNPs cocktail. Out of four NABs used, the HIV-1 gp120 antiserum (PB1 sub 2) was most potent NAB (neutralizing antibody) in inhibition of HIV-1_IIIB_. It was expected as this antibody has been raised against HIV-1_IIIB _virus. Other three heterologous NABs were found to have varying degrees of HIV-1_IIIB _inhibitory potencies. AgNPs at 1 mg/ml concentration have been shown to exert ~40% inhibition of cell free HIV-1_IIIB _virus infection. When NABs and AgNPs were used together, we recorded no additive effect of inhibition of cell free HIV-1_IIIB _Virus infection.

Inhibition of cell -associated virus infection has been found difficult to achieve by the NABs alone. A few NABs when used at high titers were able to inhibit cell associated homologous virus. We attempted to record if AgNPs will be able to increase inhibition of cell associated virus by homologous and heterologous NABs. Since cell free and cell associated both viruses are present in the infectious inoculums in real life, any increase in inhibitory potencies of NABs against HIV-1_IIIB/H9 _virus infection will be interesting. In our experiments, we have used all four NABs, described earlier, to evaluate their inhibitory effect on HIV-1_IIIB/H9 _virus infection along with AgNPs. The NABs used in this set of experiments alone did not show significant inhibition (2-10% inhibition) of cell- associated virus HIV-1_IIIB/H9 _in U373-MAGI-CXCR4_CEM _cells. The AgNPs alone however were successful in inhibiting cell associated HIV-1_IIIB/H9 _virus infection. In fact AgNPs alone were more potent (50% inhibition) with cell associated HIV-1_IIIB/H9 _virus infection than cell free HIV-1_IIIB _virus infection (40% inhibition). At present we do not know the exact reason behind increased inhibition of cell associated virus v/s cell free virus. We assume it may be due to better binding between cell associated virus and AgNPs. The use of AgNPs+ all four NABs cocktail, however, produced significant increase in inhibition of cell associated HIV-1_IIIB/H9 _virus infection. The use of this cocktail resulted in 60 to 71% inhibition of cell associated virus infection. All four antibodies used in this experiment had almost similar increase in inhibition of HIV-1_IIIB/H9 _virus infection. It appears that AgNPs when present along with NABs were able to bind different epitopes on gp120 and/or gp41 which NABs alone did not bind and vice versa. The mechanism behind this additive effect in cell-associated infection is not known and needs further evaluation. Nevertheless, this is very significant finding because cell- associated viruses are the main source of HIV-1 transmission. Recently Diane and colleagues have shown that latently infected CD4+ T cells in breast milk from women with or without antiretroviral drugs simultaneously produce HIV-1 and increase chances of transmission between mothers to infant [28]. A similar phenomenon is expected with latently infected cells in semen and vaginal secretions. In this light, the additive inhibitory efficiency of AgNPs along with NABs against cell associated virus infection is a very positive data that suggests use of this strategy in developing antiviral vaginal gel/cream to prevent HIV-1 virus transmission.

## Conclusion

The NABs have been shown to inhibit HIV-1 transmission of infection at very high titers *in vitro*. But the available vaccines under evaluation in various labs are unable to elicit such high titers *in vivo*, resulting in lowered efficacy and/or failure of vaccine against viral challenges. Silver nanoparticles used along with NABs against cell free HIV-1_IIIB _virus had no additive effect. In the case of cell associated HIV-1_IIIB/H9 _virus, all four NABs evaluated in this study showed almost no inhibitory effect by itself. Only AgNPs showed capability to inhibit cell- associated HIV-1_IIIB/H9 _virus. However, when used together, the results showed additive effect, increasing the inhibitory effect of AgNPs, and NABs cocktail in case of all four NABs used. The mechanism behind this increase in potency is not well understood and requires further study.

## Methods

### Antibodies, cells and HIV-1 isolates

The HIV-1_IIIB _virus alongwith the following reagents were obtained through the NIH AIDS Research and Reference Reagent Program, Division of AIDS, NIAID: U373-MAGI-CXCR4_CEM _from Dr. Michael Emerman, HTLV-III_B _from Dr. Robert Gallo, Monoclonal antibody to HIV-1 gp41 (126-7) from Dr. Susan Zolla-Pazner, HIV-1 gp120 Antiserum (PB1 Sub 2), HIV-1 gp120 Antiserum (PB1), and HIV-1 gp120 Monoclonal Antibody (F425 B4e8) from Dr. Marshall Posner and Dr. Lisa Cavacini.

### Silver compounds

Commercially manufactured 30-50 nm silver nanoparticles, surface coated with 0.2 wt% PVP, were used (Nanoamor, Houston, TX). Stock solutions were prepared in RPMI 1640 cell culture media. The serial dilutions of the stock were made in culture media.

### Cytotoxicity Assay

A stock solution of AgNPS was two-fold diluted to desired concentrations in growth medium and subsequently added into wells containing 5 × 10^4 ^U373-MAGI-CXCR4_CEM _cells to a final volume of 100 μl. Microtiter plates were incubated at 37°C in a 5% CO_2 _air humidified atmosphere for 24 hours. Assessments of cell viability were carried out using a CellTiter-Glo^® ^Luminescent Cell Viability Assay and Glomax Multidirection System (Promega). Cytotoxicity was evaluated based on the percentage cell survival relative to the control in the absence of any compound [21].

### Range of antiviral activity of Neutralizing Antibodies (NABs) against HIVIIIB cell-free virus

Serial two-fold dilutions of neutralizing antibodies: Monoclonal antibody to HIV-1 gp41 (126-7), HIV-1 gp120 Antiserum (PB1 Sub 2), HIV-1 gp120 Antiserum (PB1), and HIV-1 gp120 Monoclonal Antibody (F425 B4e8) or just media as control were added to HIV-1_IIIB _cell-free virus to a final volume of 50 μl. After incubation for 5 min at room temperature we added media with or without AgNPs 1 mg/mL and placed into 96-well plates with U373-MAGI-CXCR4_CEM _cells to a final volume of 50 μl. The cells were incubated in a 5% CO_2 _humidified incubator at 37°C for 24 h. Assessment of HIV-1 infection was performed with the Beta-Glo Assay System using Glomax Multidirection System (Promega). The percentage of residual infectivity after NABs or media as control was calculated with respect to the control. The 50% inhibitory concentration (IC_50_) was defined according to the percentage of infectivity inhibition relative to the positive control.

### Range of antiviral activity of Neutralizing Antibodies (NABs) against HIVIIIB cell-associated virus

Serial two-fold dilutions of neutralizing antibodies: Monoclonal antibody to HIV-1 gp41 (126-7), HIV-1 gp120 Antiserum (PB1 Sub 2), HIV-1 gp120 Antiserum (PB1), and HIV-1 gp120 Monoclonal Antibody (F425 B4e8) or just media as control were added to H9 cells (5 × 10^4 ^per well) chronically infected with HIV_IIIB _to a final volume of 50 μl. After incubation for 5 min at room temperature we added media with or without AgNPs 1 mg/mL and placed into 96-well plates with U373-MAGI-CXCR4_CEM _cells to a final volume of 50 μl. The cells were incubated in a 5% CO_2 _humidified incubator at 37°C for 24 h. Assessment of HIV-1 infection was performed with the Beta-Glo Assay System. The percentage of residual infectivity after NABs or media as control was calculated with respect to the control. The 50% inhibitory concentration (IC_50_) was defined according to the percentage of infectivity inhibition relative to the positive control.

### Statistical analysis

Graphs were done with *SigmaPlot 10.0 *software and the values shown are means ± standard deviations from three separate experiments, each of which was carried out in duplicate. Cytotoxicity and inhibition assessment graphs are linear regression curves done with *SigmaPlot *10.0 software. Wilcoxon rank-sum (Wilcoxon-Mann-Whitney test) test was performed to compare the two groups of results (HIV-1 infectivity by AgNPs, and AgNPs mixed with NABs.

## List of Abbreviations

AgNPs: Silver Nanoparticles; NABs: Neutralizing antibodies; gp120: HIV Envelop Glycoprotein 120 KD; gp41: HIV Enveloped Glycoprotein 41KD; TCID_50_: Tissue Culture Infective Dose 50; PVP: Polyvinylpyrrolidone

## Competing interests

The authors declare that they have no competing interests.

## Authors' contributions

All authors read and approved the final manuscript. HHL participated in the conception and experimental design and performed *in vitro *HIV-1 infectivity assays. He also participated in the analysis and interpretation of the data, and in writing this report. LIT participated in the conception and design of the *in vitro *HIV-1, in analysis and interpretation of the data, and in writing and revision of this report. ENG participated in *in vitro *HIV-1 infectivity assays. DKS participated in the experimental design of this research, editing and revision of this report. His lab provided materials and resources used in this study.

## Authors Information

DKS: is an associate professor of microbiology at the Winston Salem State University. DKS' lab is working on development of a DNA vaccine for HIV/AIDS. His other research interest involves prevention of HIV-1 transmission at the cervical/vaginal mucosal surfaces. His current research is funded by two NIH grants.
